# CELL SENESCENCE AND MITOCHONDRIA: A MATTER OF LIFE OR DEATH

**DOI:** 10.1093/geroni/igae098.1907

**Published:** 2024-12-31

**Authors:** Joao Passos

**Affiliations:** Mayo Clinic, Rochester, Minnesota, United States

## Abstract

Senescence is a multi-functional cell fate, characterized by an irreversible cell-cycle arrest and a pro-inflammatory phenotype, commonly known as the senescence-associated secretory phenotype (SASP). Emerging evidence indicates that accumulation of senescent cells in multiple tissues drives tissue dysfunction and several age-related conditions. This has spurred the academic community and industry to identify new therapeutic interventions targeting this process. Senescence is a complex and highly heterogenous phenotype and despite tremendous progress in recent years we are still far from fully understanding it. There is no single, stand-alone marker that allows the identification of senescent cells, posing a challenge to their identification and targeting. Mitochondrial dysfunction is an often-unappreciated hallmark of cellular senescence which plays important roles not only in the senescence growth arrest but also in the development of the SASP and resistance to cell-death. In my talk, I will first describe some of our recent efforts to accurately map senescent cells in different tissues, including the application of imaging methods involving artificial intelligence. Additionally, I will describe how mitochondria contribute to the development of senescence and resistance to cell-death. Finally, I will propose that a detailed road map of mitochondrial biology in senescence will be crucial to guide the future development of therapies targeting cellular aging.

